# Supplementary material for: Increased PIEZO1 Expression Is Associated with Worse Clinical Outcomes in Hormone-Receptor-Negative Breast Cancer Patients
Source: Cancers (Basel). 2024 Feb 6;16(4):683. doi: 10.3390/cancers16040683 (PMC10887014; doi:10.3390/cancers16040683)
Supplement: Supplementary file 1 [file cancers-16-00683-s001.zip › cancers-2806066-supplementary.pdf]

# Figure S1:

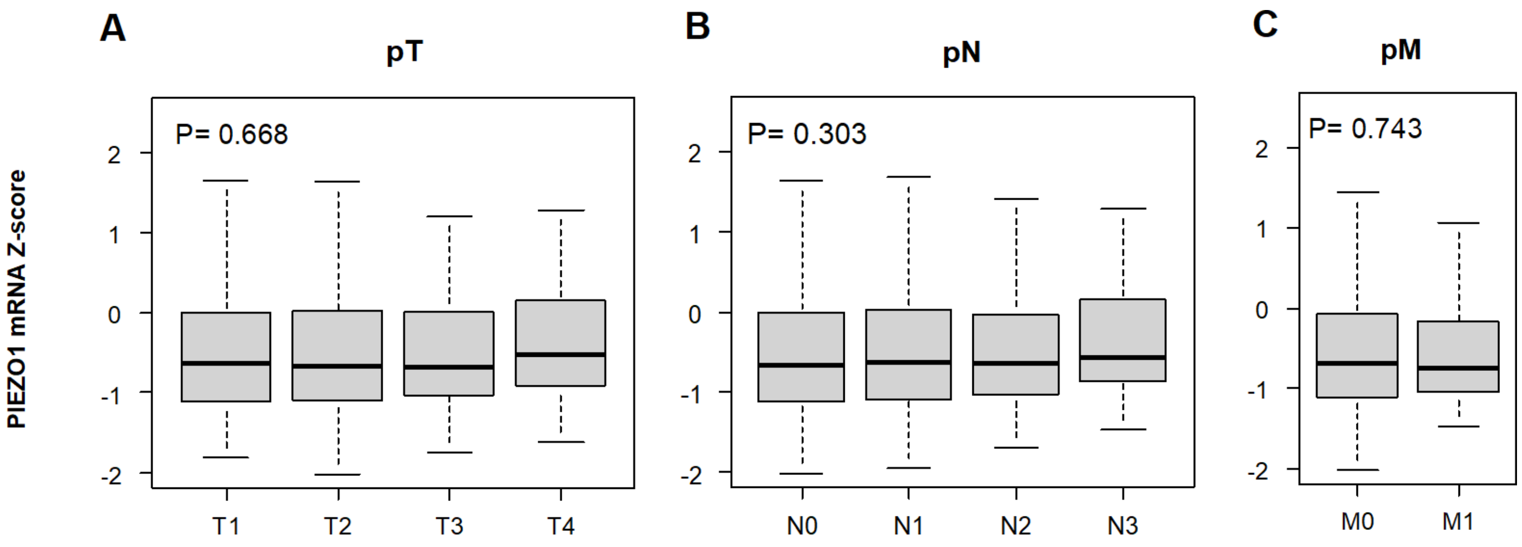


**Figure S1: PIEZO1 expression and AJCC TNM stage in TCGA breast cancer cohort. (A)** PIEZO1 mRNA expression by tumor size (pT) in TCGA breast cancer cohort. pT1: n=279; pT2: n=633; pT3: n=138; pT4: n=40. **(B)** PIEZO1 mRNA expression by spread of cancer to lymph nodes (pN) in the whole cohort. pN0: n=515, pN1; n=361, pN2; n=120 and pN3; n=77. **(C)** PIEZO1 expression levels by metastasis status (pM). pM0; n=909, pM1; n=22. Continuous value between two groups was compared by Student's t-test, and ANOVA followed by post hoc Tukey was used for comparison of more than two groups.

# Figure S2

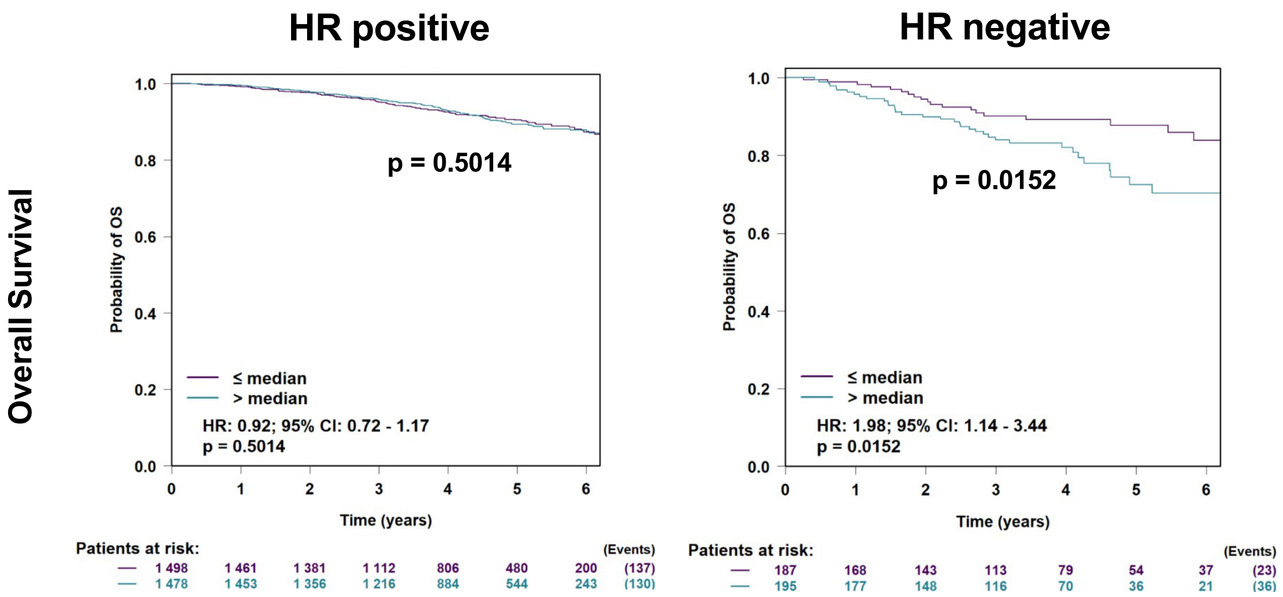


**Figure S2: Breast cancer patient survival by PIEZO1 expression in RNA-seq cohorts from bc-GenExMiner.** Overall survival by PIEZO1 expression in the HR positive and negative cohorts. Survival difference was estimated by Cox Univariate analysis.

## Figure S3

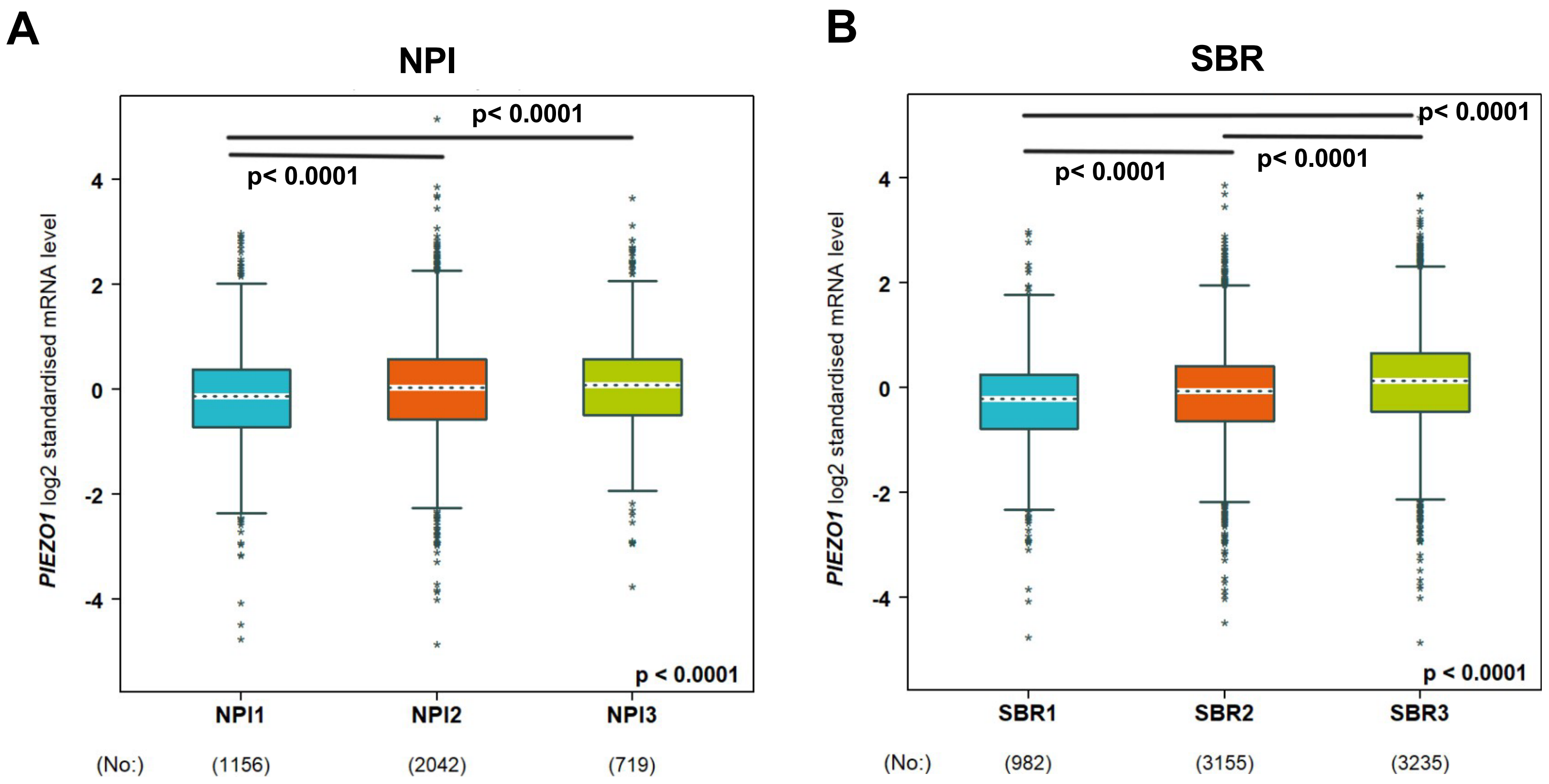


**Figure S3: PIEZO1 expression by clinical prognostic indexes from whole cohort of bc-GenExMiner. (A)** PIEZO1 expression amongst Nottingham Prognostic Index values and **(B)** amongst Scarff- Bloom-Richardson grade values, based on all DNA microarray data. Dunnett-Tukey-Kramer's test used for statistical analysis.

# Figure S4

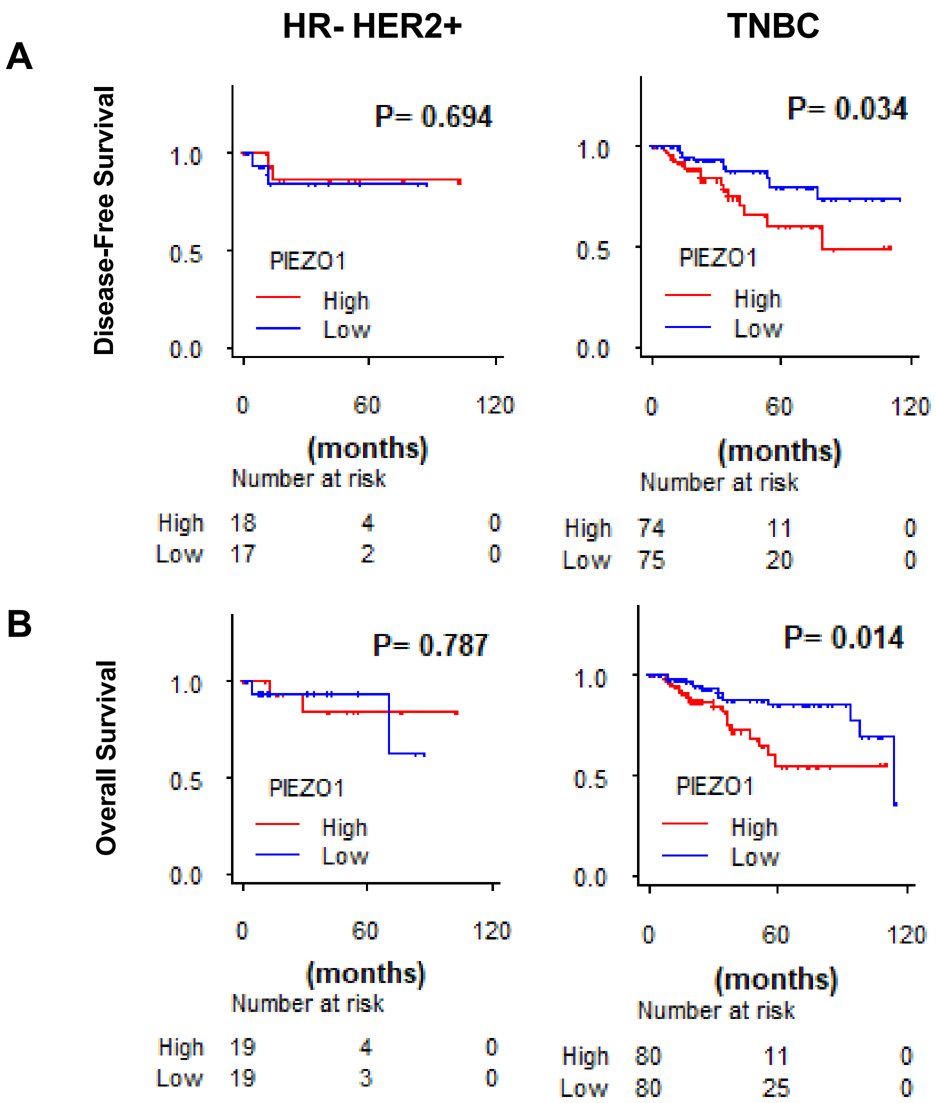


**Figure S4: Breast cancer patient survival by PIEZO1 expression in TCGA. (A)** Disease-free survival and **(B)** overall survival by PIEZO1 expression in the hormone receptor (HR) negative HER2 positive vs triple negative breast cancer (TNBC) cohorts. Survival difference was estimated by log-rank test.

## Figure S5

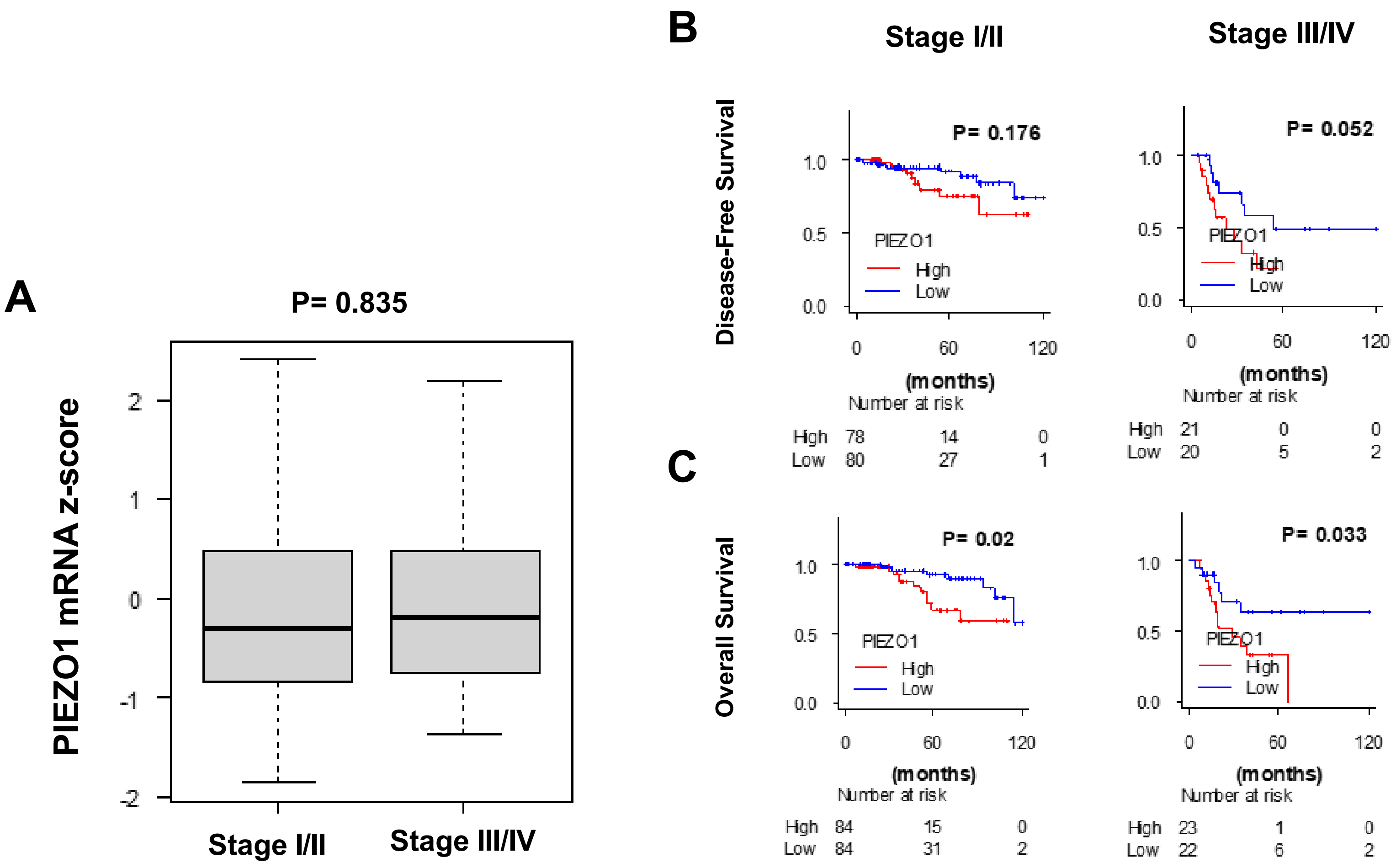


**Figure S5: PIEZO1 prognostic value by stage in HR negative breast cancer TCGA cohort. (A)** PIEZO1 expression by breast cancer stage. Continuous value between two groups was compared by Student's t-test. **(B)** Disease-free and **(C)** overall survival by PIEZO1 expression by breast cancer stage. Survival difference was estimated by log-rank test.

# Figure S6

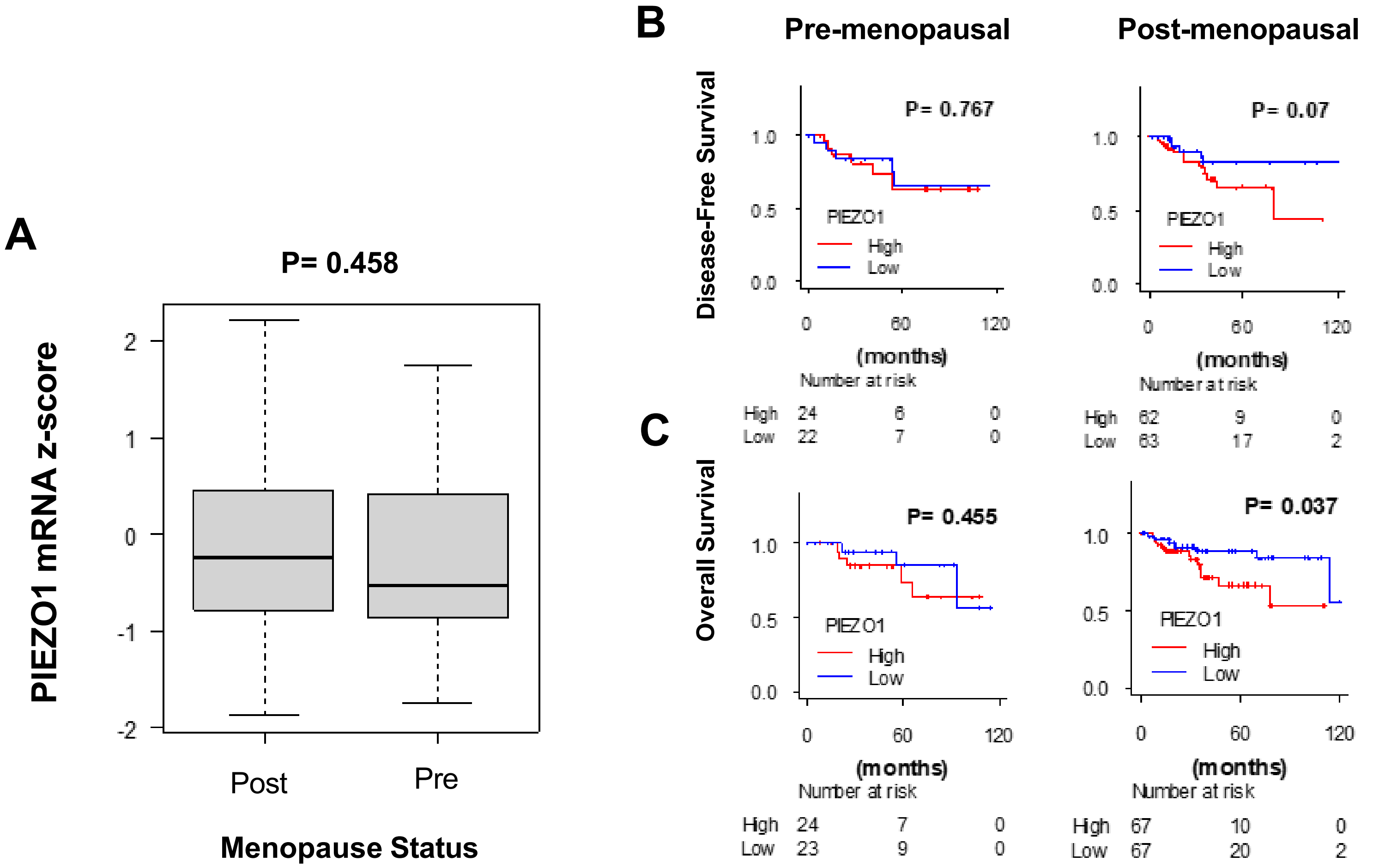


**Figure S6: PIEZO1 prognostic value by menopause status in HR negative breast cancer TCGA cohort. (A)** PIEZO1 expression in pre-menopausal vs post-menopausal patients. Continuous value between two groups was compared by Student's t-test. **(B)** Disease-free survival and **(C)** overall survival by PIEZO1 expression by menopause status. Survival difference was estimated by log-rank test.

# Figure S7

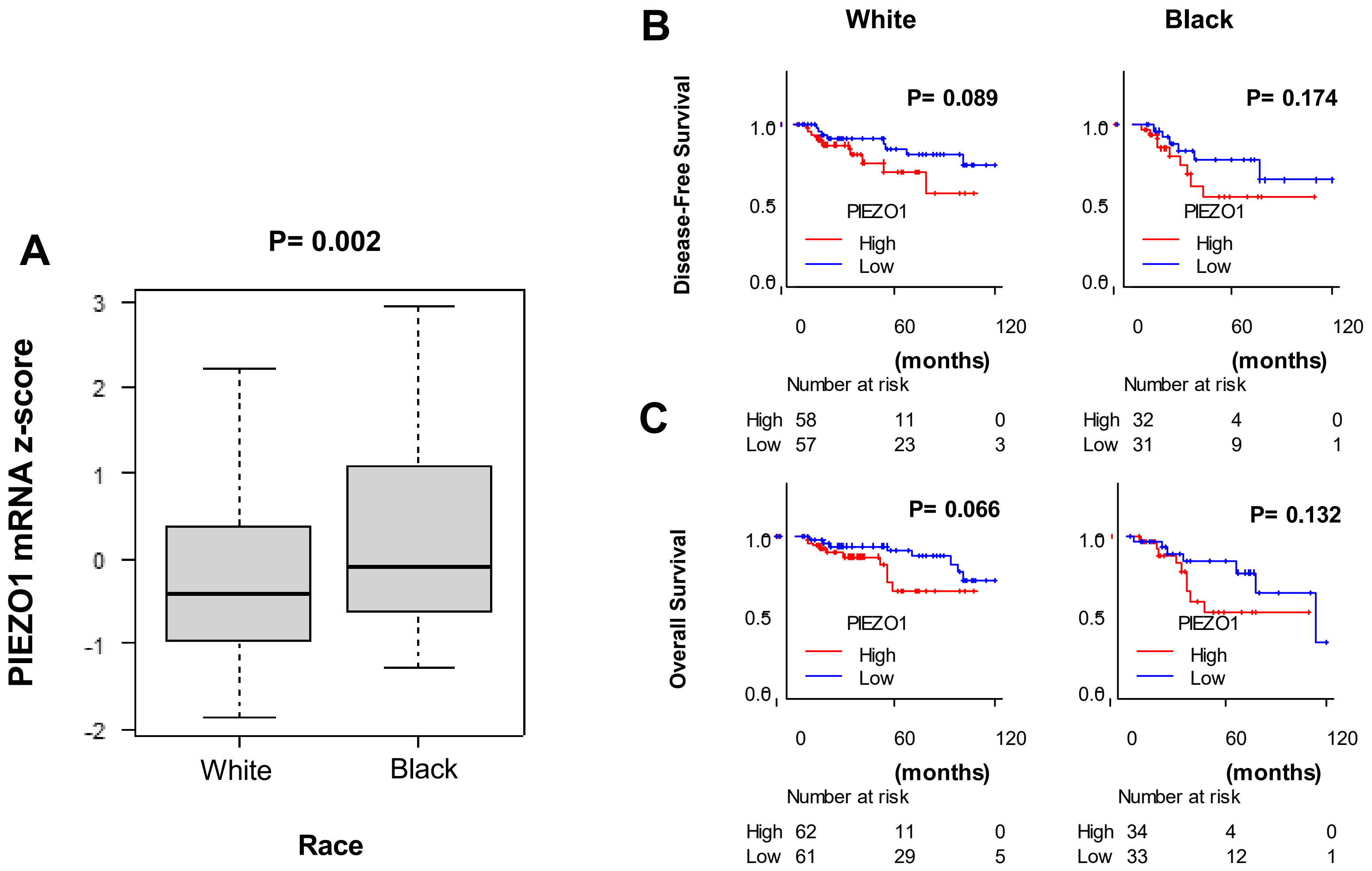


**Figure S7: PIEZO1 prognostic value by race in HR negative breast cancer TCGA cohort. (A)** PIEZO1 expression in White vs Black patients. Continuous value between two groups was compared by Student's t-test. **(B)** Disease-free survival and **(C)** Overall survival by PIEZO1 expression by race. Survival difference was estimated by log-rank test.

# Figure S8:

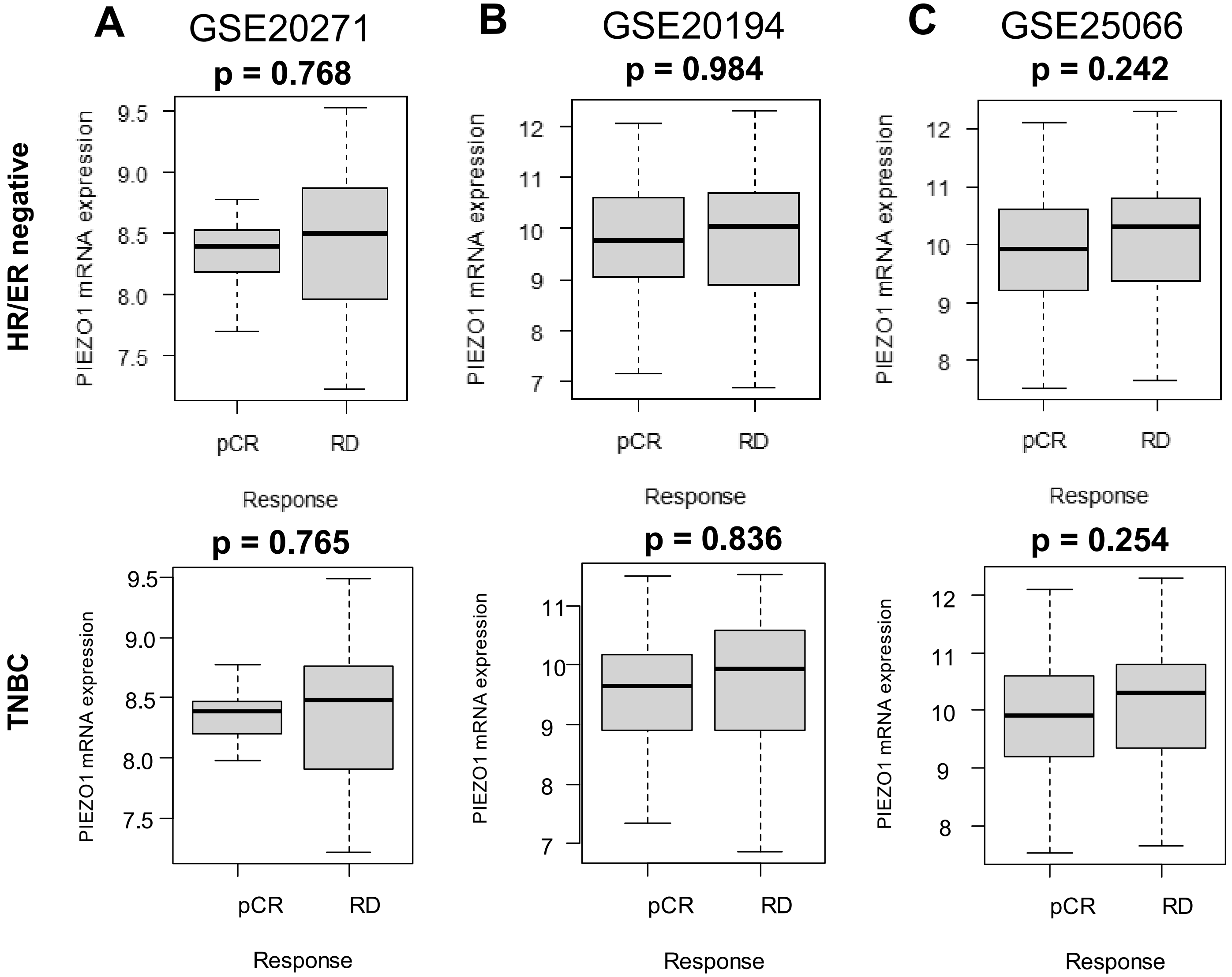


**Figure S8: PIEZO1 expression by response to neoadjuvant chemotherapy in breast cancer patients.**
**(A)** PIEZO1 expression in pre-treatment tumor by clinical response (pCR: pathological complete response; RD: residual disease) following neoadjuvant chemotherapy treatment in HR negative tumors and TNBC tumors of GSE20271, **(B)** HR negative and TNBC of GSE20194, and **(C)** ER negative and ER negative HER2 negative of GSE25066. Continuous value between two groups was compared by Student's t-test.
